# CuZn Complex Used in Electrical Biosensors for Drug Delivery Systems

**DOI:** 10.3390/ma15217672

**Published:** 2022-11-01

**Authors:** Yan Yik Lim, Azizi Miskon, Ahmad Mujahid Ahmad Zaidi

**Affiliations:** 1 Faculty of Defence Science and Technology, National Defence University of Malaysia, Sungai Besi Prime Camp, Kuala Lumpur 57000, Malaysia; 2 Faculty of Engineering, National Defence University of Malaysia, Sungai Besi Prime Camp, Kuala Lumpur 57000, Malaysia

**Keywords:** electrical biosensors, drug delivery systems, drug carriers, triggered release, stimuli, CuZn, metal of frameworks, therapeutic efficacy

## Abstract

This paper is to discuss the potential of using CuZn in an electrical biosensor drug carrier for drug delivery systems. CuZn is the main semiconductor ingredient that has great promise as an electrochemical detector to trigger releases of active pharmaceutical ingredients (API). This CuZn biosensor is produced with a green metal of frameworks, which is an anion node in conductive polymers linked by bioactive ligands using metal–polymerisation technology. The studies of Cu, Zn, and their oxides are highlighted by their electrochemical performance as electrical biosensors to electrically trigger API. The three main problems, which are glucose oxidisation, binding affinity, and toxicity, are highlighted, and their solutions are given. Moreover, their biocompatibilities, therapeutic efficacies, and drug delivery efficiencies are discussed with details given. Our three previous investigations of CuZn found results similar to those of other authors’ in terms of multiphases, polymerisation, and structure. This affirms that our research is on the right track, especially that related to green synthesis using plant extract, CuZn as a nanochip electric biosensor, and bioactive ligands to bind API, which are limited to the innermost circle of the non-enzymatic glucose sensor category.

## 1. Introduction

The electrical biosensor [1] for electrical triggers in drug delivery systems (DDS) [2] has become popular because of the advancement of nanotechnology [3,4], making the nanochip smaller than the minimum red blood cell size of 1.7 µm [5]. This nanochip [6,7,8] is used as a drug reservoir, carrier, and release to control the drug load and delivery [9]. This nanochip is usually made of conductive polymers, endogenous metals, and ligands [10,11]. The natural conductive polymers consist of hyaluronic acid, alginate, agarose, chondroitin sulphate, carbomer, and xanthan gum [12,13]. Furthermore, the synthesised conductive polymers consist of polypyrrole, polyaniline, polyacrylamide, and polymethacrylic acid derivatives [14,15]. Both conductive polymers could be hybridised into metal-organic frameworks (MOF) [16,17,18] by using ligands to bind with endogenous metals such as Ca [19], Cu [20], Zn [21], and CuZn [22]. Recently, green synthesis [23] has used bioactive ligands to coordinate the polymerisation [24] in biomedical MOFs [25] with minimum hazardous by-products [26]. These bioactive ligands [27] of saccharides, nucleases, porphyrins, peptides, or amino acids can be molecules or anions [28] that donate a pair of electrons to the central metal in the polyelectrolytes [29] during the polymerisation of a coordination complex [30,31]. Therefore, the central metal has been used as a cation nod, the chemical surface of which can be manipulated to attract most API anions [32,33]. As a result, a highly ordered crystalline nanochip could be engineered with tailored DDS features and yet be biocompatible without conventional toxicity.

Our team intended to use CuZn as the main composition of the electrical biosensor for an electrical trigger in DDS, but none have been used this way before. We believe this metallic ingredient can replace the metal in MOF to be a better electrical trigger [34] because of its better conductivity [35] as a proven semiconductor ingredient [36,37]. However, several problems, such as glucose oxidation [38,39,40], low binding affinity [41], and Cu toxicity [42], need to be solved first. The first generation of electrical biosensors is the enzyme-based amperometric biosensor [43,44], resulting in glucose-oxidised problems with a reduction–oxidation (redox) microenvironment for tumour growth [45]. This could be solved by using non-enzymatic glucose sensors (NEGS) [40,44]. The second problem is the low active pharmaceutical ingredients (API)-binding affinity, resulting in a lower API cargo carrier and a lesser choice of API variations. This could be solved by the hydrophobic grafting method through metallic surface chemical modification, or MOF [46]. The third problem is the toxicity of Cu, and this could be solved by using Zn ions in CuZn to control the toxicity [47]. Despite these problems, both Cu and Zn ingredients are always related to reactive oxygen species (ROS) [48], resulting in reduced redox reactions, glutathione (GSH), and pH values [18]. Moreover, there has been no specific discussion on CuZn being applied to the pH and redox endogenous stimuli in DDS until now. Therefore, it is rational to clarify the effects of the use of CuZn as a bioactive cation for drug loading and as an electrical trigger or with additional stimuli to release sustainability in achieving therapeutic efficacy.

**Figure 1:**
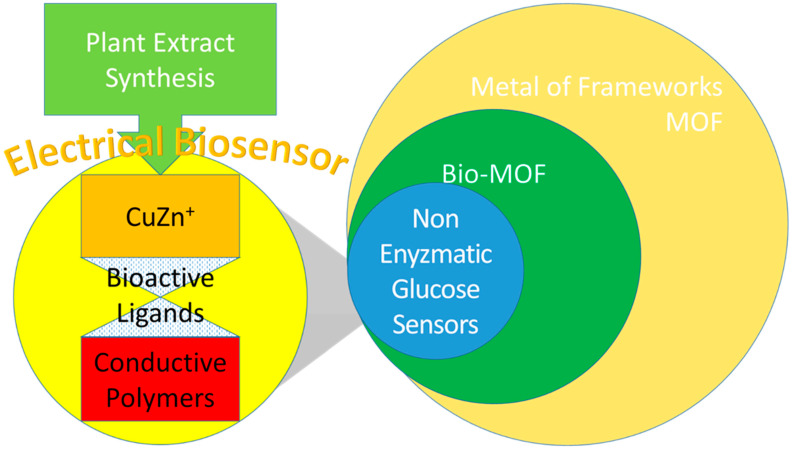
The scope of CuZn electrical biosensor design and its end-product category using plant extract synthesis as a source.

In this paper, the discussion is focused on the API that were triggered by the electrical biosensor in DDS. The aim of this paper is to elaborate on the potential of CuZn being used in the composition of electrical biosensors. Firstly, an overview of electrical biosensors being used as the electrical trigger of the API in drug carriers is presented. Then, some additional enhancements to electrical biosensors being used as the electrical triggers of the API are highlighted. Secondly, Cu and Zn were composed as the main composites in electrical biosensors, described with their significant findings. Then, the Cu composites were used as NEGS that solved the glucose oxidation problem with their electrical performance in significant findings. Following that, Cu and Zn were successfully composed into MOF that were elaborated by their electrical biosensor performance with higher API-binding affinity [49]. Thirdly, Cu and Zn were successfully used as redox stimuli and electrical biosensors for electrical triggers. Lastly, we concluded that both Cu and Zn can be used as API in chemotherapy. A diagram was drawn to describe the scope of the CuZn electrical biosensor design that should comply with the plant extract synthesis as a source and the NEGS category as the end product, as shown in Figure 1.

## 2. Electrical Biosensors for Electrical Trigger in Drug Delivery Systems

Five years ago, the synthesis of conductive polymers was the most favoured electrical biosensor used as a trigger in DDS. There are three examples of electrical triggers of API in drug carriers with biosensors and remarks, as shown in Table 1. For instance, the synthesis, polymer-like aniline tetramer (AT), was used by Atoufi et al. (2017) as an electrical biosensor in an alginate (Alg)/agarose/AT drug carrier to release dexamethasone (Dex) [50]. They found that the 10% aniline tetramer sample had the highest ionic conductivity and was suitable for neuroregenerative medicine. Later, Lestari et al. (2018) used Zn as an electrical biosensor in a drug carrier containing benzene 1,3,5-tricarboxylate (BTC)_2_/Zn_3_ to release ibuprofen [51]. They synthesised a MOF for BTC_2_/Zn_3_ drug carriers using a green synthesis method such as electro-sonochemistry at 15 V and 40 KHz. Their significant findings showed a slow release or sustainable delivery of the API. Another MOF was produced by Wang et al. (2018) using Cu as an electrical biosensor in an Au@Cu drug carrier to release microRNA (miR)-155 [52]. The biosensor performance was detected by miR-155 concentrations ranging from 1.0 fM to 10 nM, which was the sensitivity of glucose oxidation measured by differential pulse voltammetry (DPV) responses. The detection limit was found to be 0.35 fM, so the ratio of signal-to-noise was three-fold. As a result, the electrical biosensor showed a highly sensitive detection of miR-155. Forero et al. (2017) [53] used an electrical biosensor of CuZn, which was better than either Zn by Lestari et al. (2018) or Cu by Wang et al. (2018). They made a bone tissue engineering scaffold with the appropriate pore size, interconnection, and high porosity that was similar to that of our studies [54]. Natural API such as hydroxyapatite (HAp) was used in the CuZn/gelatin/chitosan (Chi) drug carrier for bone repair and regeneration, including mineralisation, collagen stimulation, osteoblast cell adhesion, and proliferation. The results showed them to be suitable for a bone tissue regeneration scaffold because the alkaline phosphatase (ALP) activity as an osteogenic differentiation marker increased the antibacterial activity of osteoblasts and non-toxicity for osteoprogenitor cells.

The conductive polymers used in electrical biosensors in DDS are more advanced, such as AT as a synthesis polymer, for which it was found that the 10% AT sample had the highest ionic conductivity in an Alg/agarose/AT drug carrier. Another Zn electrical biosensor was improved by a green synthesis method of electro-sonochemistry in a BTC_2_/Zn_3_ drug carrier. Glucose oxidation is the main problem of electrical biosensors, the miR-155 release of which is measured by DPV responses. Therefore, a Cu electrical biosensor in a Au@Cu drug carrier was used to measure its sensitivity and found its detection limit at 0.35 fM. Lastly, we managed to find an electrical biosensor using CuZn in a CuZn/gelatinChi drug carrier that was similar to our aim of making a CuZn electrical biosensor. Although, these findings showed an ALP activity increment and were non-toxic to osteoprogenitor cells, which were suitable for the bone tissue engineering scaffold. This was similar to the three previous findings, in which no glucose oxidation test was conducted. As a result, it is still too early to conclude that CuZn has the potential to be an electrical biosensor in DDS.

### Additional Features Enhancements of Electrical Biosensors for Electrical Triggers

At present, scientists and researchers demand more functions from the metal implanted into drug carriers. There are five enhancement examples of electrical biosensors for electrical triggers of API in drug carriers with remarks, as shown in Table 2. The first two examples used iron oxide nanoparticles (IONPs) in MOF for drug carriers in order to obtain electrical triggers for DDS and magnetic-field-targeted delivery. For instance, Yu et al. (2020) used IONPs as magnetic targetors and an electrical biosensor to deliver and release indoleamine 2,3-dioxygenase inhibitors (IDOis) in an IONP/IDOi drug carrier [55]. The electric pulse created irreversible electroporation, resulting in a local magnetic field that had a synergistic effect on immuno-ablation cancer therapy. These significant findings showed that T-cell regulation was increased by CD8^+^ T-cell infiltration and its ratio to eliminate primary and secondary tumours. Furthermore, Mohapatra et al. (2020) used polyethylene glycol dimethacrylate (PEGDMA) as an electrical biosensor and an IONP as a magnetic targetor to release vancomycin (Vcm) in an IONP/Chi/PEGDMA drug carrier [56]. The electrical triggers were generated by 100 Hz bipolar electric pulses, the ion distributions and migrations of which were studied by COMSOL stimulation software. The significant findings showed the electric stimulation was controlled and the targeted implanted site was delivered. The third and fourth studies using Cu as an electrical biosensor in the MOF drug carriers were performed by Gharehdaghi et al. (2022) [57]. The main biological structure of BTC in the third drug carrier was similar to that of Lestari et al. (2018) [51], but the Zn biosensor was replaced with Cu and an additional IONP magnetic targetor. The fourth drug carrier also had Cu as an electrical biosensor, but the IONPs and BTC were replaced with graphene oxide (GO) [58] and tetrakis (4-carboxyphenyl) porphyrin (TCPP), respectively. Both BTC and TCPP have pH-sensitive carboxy functional groups that after 60 h of testing released doxorubicin (Dox) of 33.5% at pH 7.4, but released 85.5% and 98.9% at pH 5, respectively. The difference was that the IONPs and GO were used for magnetic and photothermal stimuli in DDS, respectively. Cu_3_(BTC)_2_ was an octahedron network structure that had a higher drug loading capacity than the Cu-TCPP crystals that were embedded between exfoliated GO layers. However, the GO/Cu-TCPP drug carrier adsorbed a total Dox of 45.7 wt.%, which was higher than that of the IONP/Cu_3_(BTC)_2_ drug carrier of 40.5 wt.%. As a result, the GO/Cu-TCPP drug carrier was better because of its higher release in response to lower pH and higher drug adsorption. The lower drug loading capacity compared with IONP/Cu_3_(BTC)_2_ could be improved by the surface chemical modification of GO. The fifth example was an enhancement of a drug reservoir, which was unsealed to release API by an electrical trigger. Patil et al. (2020) studied transdermal DDS efficiency using pullulan (Plt)/poly(vinyl alcohol) (PVA) and polyacrylamide (polyAAm)/Plt/PVA as an electrical biosensor and a drug carrier, respectively [59]. They discovered an electrical trigger from 2 to 8 mA that increased the diffusion rate of rivastigmine tartarate (RT) by 1.68-fold. Even though RT is used for Alzheimer’s disease, it can be replaced with API such as capsaicin, salicylates, counterirritants, and anaesthetics used for rheumatoid arthritis therapies. This drug reservoir could also be converted to a nanochip for API-triggered release when connected to currents of about 8 mA or electric pulses of around 100 Hz.

The first two drug carriers with magnetic enhancements used IONP and PEGDMA biosensors to release IDOi and Vcm, which eliminated tumours through T-cell regulation and targeted delivery with controlled release, respectively. The following two drug carriers with magnetic enhancements of IONPs and GO as photothermal stimuli stimulated the carboxy functional groups at pH 5 to release Dox of 85.5% and 98.9%, respectively. However, GO/Cu-TCPP was better because of its 45.7 wt.% drug adsorption, which was higher than the 40.5 wt.% of IONP/Cu_3_(BTC)_2_. Finally, another different concept of enhancement is a drug reservoir in a polyAAm/Plt/PVA drug carrier for transdermal therapy. This Plt/PVA was used as an electrical biosensor and reservoir, and it was found that the RT diffusion rate increased by 1.68-fold when connected with a current of 2 to 8 mA. This discovery has driven us to design a nanochip using CuZn as a drug reservoir.

## 3. Cu and Zn as the Main Composites in Electrical Biosensors for Electrical Triggers

### 3.1. Cu in Non-Enzymatic Glucose and Electrical Biosensors

While designing an electrical biosensor for the electrical trigger of API, the main problem is glucose oxidation, which reduces oxygen during the electrolytic process in the body. This oxygen reduction is redox, similar to bacteria consuming oxygen in a tumour microenvironment (TME). The glucose oxidation process [60] and the acid chemical reaction are described in Equations (1) and (2), respectively:

(1) $$C_{6}H_{12}O_{6}+6O_{2}\to 6{\mathrm{CO}}_{2}+6H_{2}O+\mathrm{Energy}$$

(2) $${\mathrm{CO}}_{2}+H_{2}O\to {\mathrm{HCO}}_{3}^{-}+H^{+}$$

where C_6_H_12_O_6_, O_2_, CO_2_, H_2_O, HCO_3_^−^, and H^+^ are glucose, oxygen, carbon dioxide, water, bicarbonate, and hydrogen ions, respectively.

The general mechanism of glucose oxidation is glucose being oxidised to produce carbon dioxide, water, and entropy energy. The carbon dioxide and water are further reacted to bicarbonate and hydrogen ions to form an acidic condition. Thus, the acidic and redox microenvironments are TMEs, so the enzymatic glucose sensor using glucose oxidation mechanisms is not an optimal choice. Therefore, NEGS made of Co, Ni, or Cu electrodes should be used because they generate current without redox reactions and acidic conditions. Three examples of Cu were used as NEGS with their findings shown in Table 3. For instance, Yao et al. (2016) composed an electrode with Cu nanoparticles (NPs) and a multi-walled carbon nanotube (MWCNT) that used an electrical biosensor of hydroquinone (HQ) release for the electrochemical performance analysis [61]. The biosensor performance was detected by the cyclic voltammetry linear response (CVLR) that was found to be as low as 0.04 μM of the detection limit at HQ concentrations ranging from 0.10 to 100 μM. Even though they found a synergistic effect on the electrochemical signal increments from the Cu-MWCNT drug carrier, the interference experiment with the glucose concentration test was not conducted to determine whether it was a NEGS. Furthermore, Zheng et al. (2016) composed an electrode with Cu, polyaniline (PANI), and GO electrical biosensors, the electrochemical performance of which was also analysed with CVLR at 0.5 V [62]. The findings demonstrated the best performance at 1% GO composite for this electrochemical glucose sensor that had high sensitivity at the linear fitting slope of 0.15 Acm^−2^M^−1^ and a detection limit of 0.27 µM at glucose concentrations ranging from 1.0 to 960 μM. Additional glucose tests using interference of either ascorbic acid or dopamine found no electrochemical signal, indicating that glucose oxidation resulting from a dedox microenvironment had not occurred. As a result, this Cu/PANI/GO composite demonstrated electrochemical conductivity and great potential to be a NEGS. Yang et al. (2020) composed an electrode with Cu, poly(3,4-ethylenedioxythiophene) (PEDOT), and phytic acid (PA) as an electrical biosensor, the CVLR electrochemical performance of which was found at 0.55 V [63]. Their findings showed that this electrochemical glucose sensor had a fast response time of 4 s, was highly sensitive with a slope of 79.27 Acm^−2^M^−1^, and had a detection limit of 0.28 µM at glucose concentrations ranging from 5 to 403 μM. As a result, this Cu/PEDOT/PA composite demonstrated an electrochemical conductivity that was similar to the two composites mentioned above, having great potential to be a NEGS.

### 3.2. Cu in MOF Compositions for Electrical Biosensors

Those Cu composites mentioned above could be bound into MOF in order to be drug carriers for API loading and release, as shown in Table 4. For instance, Sattar et al. (2016) composed three types of Cu MOF drug carriers, such as copper serinate (CS), copper prolinate (CP), and copper threoninate (CT), to release rosuvastatin (Rsv) [64]. The significant findings for drug adsorptions (DAs) by the high-performance liquid chromatography (HPLC) parameters were 0.25, 0.15, and 0.25 g/g for CS, CP, and CT, respectively. Moreover, Sattar & Athar (2018) further studied CS, CP, and CT by the HPLC parameters to release API such as terazosine hydrochloride (TH), telmisartan (Tms), and glimpiride (Glp) [65]. These significant findings of DAs for the corresponding CS, CP, and CT drug carriers showed that TH, Tms, and Glp released 0.095, 0.200, and 0.316 g/g; 0.083, 0.160, and 0.085 g/gs; and 0.041, 0.138, and 0.138 g/g, respectively. Another Cu MOF was composed by Mohammadhassan et al. (2022) using CuO and bovine serum albumin (BSA) [66]. This CuO/BSA composite had an 8.70% loading efficiency and a 75% release of methotrexate (Mtx) in the presence of enzymes at pH 7.4 after 96 h. These findings showed cytotoxicity in the MDA-MB-231 cell line and faster Mtx release in the presence of proteinase K. As a result of these three studies, it can be concluded that a Cu MOF is suitable to be used for drug loading and release in DDS.

### 3.3. Zn in MOF Compositions for Electrical Biosensors

The Zn element can also bind into MOF like Cu to be drug carriers for API loading and release, as shown in Table 5. For instance, Azizi Vahed et al. (2019) composed a drug carrier with zeolitic imidazolate frameworks (ZIF-8) or Zn(CH_3_CO_2_)_2_ and alginate (Alg) to release metformin (Mfm) [67]. ZIF-8 is a MOF that is composed of Zn ions and imidazolate ligands. The findings showed that this composite had large pore sizes of 11.6 Å, a loading efficiency of 83.5%, and a drug payload of 6.68 wt.%. As a result, the Mfm release was effectively controlled in the presence of the zinc ions. Furthermore, Zhang et al. (2019) also composed a drug carrier using ZIF-8, but replaced Alg with hyaluronic acid (HA) to release tetracycline (Tet) for a synergistic intracellular bacteria elimination system [68]. Their findings demonstrated an over 98% Tet clearance rate under acidic or pH-responsive conditions. As a result, both Tet and Zn were antibiotics that could be optimised for their efficacy–dosage correlation in an intracellular bacteria elimination system. Fu et al. (2020) composed a similar ZIF-8/HA drug carrier, but the API release was chlorin e6 (Ce6) [69]. Ce6 is a second-generation photo-therapeutic dye used for photodynamic therapy (PDT). The findings showed that ROS induced by PDT killed the HepG2 cells by about 88.4%, resulting in a cytotoxicity increment, and the systemic toxicity was reduced. As a result, this ZIF-8/HA drug carrier once again showed its therapeutic efficacy, biocompatibility, and drug deliverability. Sathishkumar et al. (2021) used ZnO to compose a different MOF drug carrier to release quercetin (Qct) [70]. This ZnO/Qct drug carrier had a size ranging from 21 to 39 nm with a hexagonal shape, which was stable under physiological pH, indicating no degradation. The significant findings were high biocompatibility with 3T3-L1 cells and effective MCF-7 growth inhibition. As a result of these four studies, Zn MOF was determined to be suitable for drug loading and release in DDS.

The electrochemical performance of Cu and Zn electrical biosensors to be used as electrodes was analysed by CVLR. Firstly, three Cu electrical biosensor electrodes composed of MWCNT, PANI, and GO, and with PEDOT and PA were found to have synergistic effects on electrochemical conductivity with their detection limits at 0.04, 0.27, and 0.28 µM for the concentration range of 0.10 to 100 μM, 1.0 to 960 μM, and 5 to 403 μM, respectively. Moreover, Cu/PANI/GO and Cu/PEDOT/PA composites both demonstrated great potential as NEGS, with a linear fitting slope sensitivity of 0.150 and 79.27 Acm^−2^M^−1^, respectively. Secondly, three Cu MOF electrodes of CS, CP, and CT were used as electrical biosensors to release Rsv, and their HPLC parameters were found to be 0.25, 0.15, and 0.25 g/g, respectively. After that, these three electrodes were used again to release TH, Tms, and Glp, and their HPLC parameters were found to be 0.095, 0.200, and 0.316 g/g; 0.083, 0.160, and 0.085 g/g; and 0.041, 0.138, and 0.138 g/g, respectively. Thirdly, the electrochemical performance of ZIF-8 electrodes with either Alg or HA composites as electrical biosensors to release Mfm, Tet, or Ce6 was analysed. The ZIF-8/Alg and ZIF-8/HA drug carriers were found to have a 6.68 wt.% drug payload for 83.5% loading efficiency and a 98% clearance rate with about 88.4% cell death after the ROS increment, respectively. Lastly, the CuO and ZnO MOF electrodes in the CuO/BSA and ZnO/Qct drug carriers’ performances were found to have an 8.7% loading efficiency for 75% Mtx release in the enzyme and an effective MCF-7 growth inhibition, respectively. Both Cu and Zn were successfully encapsulated into MOF to deliver API, as shown in Table 4 and Table 5. As a result, all Cu and Zn or their oxides in MOF demonstrated conductivity, biocompatibility, therapeutic efficacy, and drug delivery efficiency. Both Cu and Zn are metallic materials, so a Zn MOF should be similar to a Cu MOF and should ideally be NEGS [71], as shown in Table 3. However, this assumption has not yet been proven by experimentation. Therefore, our team will continue to investigate CuZn as the semiconductor’s main component in electrical biosensors to trigger and control API release in DDS.

## 4. Other Stimuli and Chemotherapies

### 4.1. Cu and Zn for Redox and pH Stimuli in Electrical Biosensors

There are many studies on Cu and Zn regarding the ROS that reverses redox microenvironments. However, there are not many studies about their use as redox stimulus biosensors. Therefore, some usages of redox and pH biosensors for endogenous stimuli are highlighted below. For instance, de Vries et al. (2022) used Zn and β-diketiminate (BDI) to create a redox stimulus coordination complex [72]. In this complex, Zn was an inert metal ion (Zn^2+^) linked to the BDI catalyst by lactide ring-opening polymerisation (LROP) to form a zinc formazanate ligand polymer (Zn/BDI). Their findings showed that this polymer was a reversible redox stimulus, but drug release tests were not conducted. Sun et al. (2020) used Cu, carbon dots (CD), and Ce6 to create a Cu/CD/Ce6 drug carrier [73]. The first finding showed that when Ce6 was activated by near-infra-red (NIR) irritation, GSH increased and pH decreased to an acidic condition. The second finding showed Cu reacting with hydrogen peroxide (H_2_O_2_) and GSH to increase the ROS in a TME. The last finding showed that the created Cu^2+^ ions were involved in chemodynamic therapy. As a result, these three findings showed that Cu/CD/Ce6 constituted a synergistic trimodal cancer therapy that was photothermal, photodynamic, and chemodynamic. As far as we know, there is still no study using CuZn as a redox stimulus biosensor.

**Figure 2:**
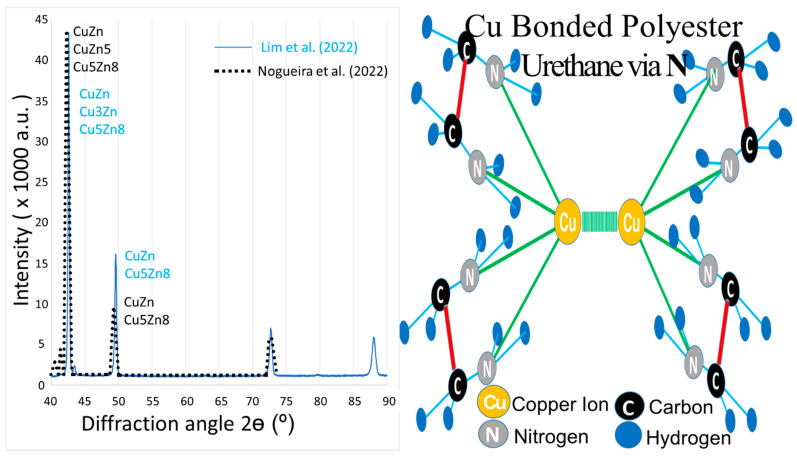
XRD intensity against diffraction angle of 2θ for CuZn (left) [74,75] and illustration of Cu-bonded polyester urethane via N (right) [54]. Reprinted with permission.

Recently, our X-ray diffraction (XRD) tests [74] for CuZn powder found that the results were coincidently similar to those of Nogueira et al. (2022) [75], as shown on the left of Figure 2 with the blue colour lines and black colour dots, respectively. The XRD test found two main peaks at two similar diffraction angles of 2θ degrees, indicating the CuZn and Cu_5_Zn_8_ phases. In another study of ours, the multi-phases of 70Cu30Zn and 80Cu20Zn were further investigated for their fracture mechanisms [76]. Although the relationship between the fracture mechanisms and residual membrane stresses was established, the relationship between the multi-phases of Cu_x_Zn_y_ and the multivalent metal ion pairs containing Cu^3+^/Cu^2+^/Cu^+^ and Zn^3+^/Zn^2+^/Zn^+^ was not. These multivalent ions are crucial for the human body’s biocatalytic reactions [46]. Furthermore, Cu bonded with polyester via nitrogen (N) to form a LROP structure, which was illustrated in our previous study of structural characterization, as shown at the right of Figure 2 [54]. Thus, this Cu had a similar function to the inert metal ion (Zn^2+^) that was linked by an active ligand in LROP. As a result, Cu has the potential to replace Zn, and further, CuZn could also replace Zn in LROP to create a drug carrier for DDS.

### 4.2. Cu and Zn as API in Chemotherapy

Cu with an NP size ranging from 1 to 100 nm has recently been found to have great potential in copper-lowering therapy for antibodies, such as angiogenesis, fibrotic, and inflammatory ones. Zn has an NP size of 0.139 nm and antioxidant properties that have been used for many medical purposes for many years. Despite its multi-therapeutical efficacy, Zn is also used for a genetic disorder called Wilson’s disease that controls excess copper built up in the body [47]. Therefore, we believe that Cu and Zn combinations could be used for chemotherapy and have potential as biosensors in DDS. However, the oxidation problem of Cu should be taken into consideration for safety and pharmacological effects. For instance, CuO NP with a high concentration may suppress the human immune system by inflicting toxicity, resulting in cell death on lymphocytes. The recent advances in coating, encapsulating, pro-drug, and MOF technologies enable us to stabilise the pharmacokinetics and pharmadynamics of CuO NP. Another choice is using plant-synthesized CuO, which showed pharmacological effects in tumour therapy, such as apoptosis and autophagic cell death, ROS increase, and metastasis inhibition [77]. This process using plant extract synthesis and microorganisms is a green MOF synthesis that does not produce toxic by-products [78]. For Zn, it is oxidised to ZnO, which does not have any toxic problems. Moreover, ZnO NP is an antimicrobial and anticancer-like genotoxic drug [79], which is also suitable as an anti-osteosarcoma scaffold [80]. Because of their high conductivity, Cu, Zn, and CuZn could be synthesised through physical, chemical, biological, and hybrid techniques [81]. They have high conductivities for lighting, heating, and electricity, making them suitable to be biosensors in DDS for photo, thermal, and electrical stimulations, respectively.

In summary, the Cu/CD/Ce6 drug carrier was discovered that Cu^2+^ had synergistic therapeutic effects on photo and chemodynamic therapy that were increased by ROS and Ce6 for redox and photothermal stimuli, respectively. Moreover, de Vries et al. (2022) created a Zn/BDI redox stimulus complex with LROP that was similar to our proposed structure of CuZn/polyester urethane in our structural characterization studies. Our XRD results for CuZn were also similar to those of Nogueira et al. (2022), who found two main peaks at two diffraction angles of 2θ degrees, indicating the CuZn and Cu_5_Zn_8_ phases. As a result, CuZn has great promise to be used as a multiple biosensor in drug carriers. Regarding the chemotherapy issue, Cu has great potential in copper-lowering therapy, which is coupled with Zn for Wilson’s disease to control excess Cu. CuO is also safe and pharmacological after coupling with ZnO to balance the suppression of the human immune system. As a result, CuZn composites were produced by advanced synthesis techniques to be biosensors with a good combination of chemotherapy, pharmacokinetics, and pharmadynamics for use in DDS.

## 5. Conclusions

Three composites including Zn, Cu, and CuZn in drug carriers each had the potential to be electrical biosensors to release ibuprofen, miR-155, and HAp, respectively. Some drug carriers were enhanced for magnetic-targeted delivery, photothermal stimuli, and electrical drug reservoirs, respectively. Three Cu-composed electrodes found their electrochemical performances at detection limits ranging from 0.04 to 0.28 µM, with two showing great promise of linear sensitivity to be NEGS. The MOF composed of Cu, CuO, and Zn had a loading efficiency ranging from 4.1% to 31.6%, 8.7%, and 83.5%, respectively. They also demonstrated biocompatibility, therapeutic efficacy, and drug delivery efficiency. The Zn/BDI drug carrier for the redox and photothermal stimuli had a LROP structure that was similar to our proposed CuZn/polyester urethane composite structure, resulting from the CuZn and Cu_5_Zn_8_ multi-phases. Moreover, the Cu/CD/Ce6 drug carrier showed photodynamic and chemodynamic therapies. Cu and Zn each had multi-therapeutic efficacy, and their combination had synergistic efficacy in chemotherapy. CuZn should become available for electrical biosensors because of pharmaceutical or green technology advancements to make them pharmacologically, pharmacokinetically, and pharmadynamically safer.

## Figures and Tables

**Table 1:** Electrical biosensors for the electrical trigger of API in drug carriers with remarks.

Biosensor	API	Drug Carrier	Remarks	Ref.
AT	Dex	Alg/agarose/AT	Conductivity and suitable for neuroregenerative medicine.	[50]
Zn	Ibuprofen	BTC_2_/Zn_3_	Electro-sonochemitry MOF at 15 V, 40 KHz with a slower release function.	[51]
Cu	miR-155	Au@Cu	DPV detection limit of 0.35 fM, so highly sensitive detection of miR-155.	[52]
CuZn	HAp	CuZn/gelatin/Chi	Increased antibacterial activity and non-toxicity for osteoprogenitor cells.	[53]

**Table 2:** Electrical biosensors with additional features for electrical triggers in drug carriers with remarks.

Biosensor	API	Drug Carrier	Remarks	Ref.
IONPs	IDOi	IONP/IDOi	Synergistic electric pulse and local magnetic field effects on immuno-ablation cancer therapy	[55]
PEGDMA	Vcm	IONP/Chi/PEGDMA	Stimulated control release and targeted delivery	[56]
Cu	Dox	IONP/Cu_3_(BTC)_2_	API released 85.5% at pH 5 and adsorbed API with 40.5 wt.%.	[57]
Cu	Dox	GO/Cu-TCPP	API released 98.9% at pH 5 and adsorbed API with 45.7 wt.%.	[57]
Plt/PVA	RT	polyAAm/Plt/PVA	Efficient reservoir for transdermal DDS	[59]

**Table 3:** Cu in electrodes of biosensors for their NEGS with the findings.

Cu Biosensor	Findings	Ref.
Cu-MWCNT	Increased electrochemical signals with a detection limit of 0.04 μM.	[61]
Cu/PANI/GO	A sensitivity of 0.15 Acm^−2^M^−1^ with a detection limit of 0.27 µM.	[62]
Cu/PEDOT/PA	A sensitivity of 79.27 Acm^−2^M^−1^ with a detection limit of 0.28 µM.	[63]

**Table 4:** Cu in a MOF for electrodes of electrical biosensors in drug carriers with the findings.

Drug Carrier	API	Findings with Slow Release	Ref.
CS, CP, and CT	Rsv	Corresponding carriers with DAs of 0.25, 0.15, and 0.25 g/g.	[64]
	TH, Tms and Glp	Corresponding carriers with DAs of 0.095, 0.200, and 0.316 g/g TH; 0.083, 0.160, and 0.085 g/g Tms; and 0.041, 0.138, and 0.138 g/g Glp.	[65]
CuO/BSA	Mtx	Drug loading efficiency of 8.70% and 75% release in proteinase K enzyme at pH 7.4.	[66]

**Table 5:** Zn in a MOF for electrodes of electrical biosensors in drug carriers with the findings.

Drug Carrier	API	Findings	Ref.
ZIF-8/Alg	Mfm	11.6 Å pore size, 83.5% loading efficiency, and 6.68 wt.% payload.	[67]
ZIF-8/HA	Tet	98% Tet clearance rate under acidic conditions and pH-responsive.	[68]
ZIF-8/HA	Ce6	88.4% of HepG2 cell death was by ROS.	[69]
ZnO/Qct	Qct	High biocompatibility with 3T3-L1 cells and effective MCF-7 growth inhibition.	[70]

## Data Availability

Not applicable.
